# The occurrence of an additional (accessory) lobe of liver and undescended testis in a single cadaver: a case report

**DOI:** 10.1186/s13256-019-2294-2

**Published:** 2019-12-06

**Authors:** Dawit Habte Woldeyes

**Affiliations:** Anatomy unit, Department of Biomedical Sciences, College of Medicine and Health Sciences, Bahir Dar University, P.O.box 79, Bahir Dar, Ethiopia

**Keywords:** Liver, Accessory lobe, Undescended testis, Testicular descent, Variations

## Abstract

**Background:**

Anatomical variations are common, some of these variations are clinically important and some are not. These variations may require treatment or they may be a variant of a normal presentation. In clinical practices, anatomical variations should not be overlooked. Anatomical variations may cause a tendency to some diseases, and may affect the symptoms, diagnosis and the course of disease. The main objective of this case report is to present the occurrence of two variations observed in a single cadaver. Even though there are reports of individual cases, these combined variations have not been reported before. There is also no evidence of developmental (embryological) circumstances for the liver anomaly to be associated with an undescended testis (cryptorchidism) and vice versa; therefore, this case is, by far, a coincidence.

**Case presentation:**

The two anatomical variations were noticed in an unclaimed male cadaver used for routine teaching and learning purposes. The Amhara male cadaver was approximate 41-year-old and his clinical history, family history, and other details were unknown. In the first incident, unusually the cadaver’s liver consisted of one additional (accessory) lobe situated on the visceral surface of the liver. In the second incident, an undescended testis was observed on the right side near to the superficial inguinal ring.

**Conclusions:**

Overall, knowledge of the above-mentioned anatomical variations has clinical significance to students, researchers, clinicians, surgeons, and radiologists who interpret plain and computed imaging.

## Background

The liver is the largest gland and the second largest organ of the body. It is situated under the right dome of the diaphragm and mainly occupies the right hypochondriac and epigastric regions. Under normal circumstances, it is divided into anatomical right and left lobes by the falciform ligament on the diaphragmatic surface, as well as by the fissure for ligamentum venosum and ligamentum teres on the visceral surface. It also has caudate and quadrate lobes as parts of the right anatomical lobe [1, 2]. In disease conditions, there is variation in liver size and morphology. Variations in the orientation are also seen in an individual as a result of body build. This makes it difficult to accurately assess the liver size by manual palpation as is done in clinics [3].

The liver is exposed to a number of congenital anomalies. The major anomalies of the liver have been classified as accessory liver lobe and ectopic liver tissue [4]. Deformed lobe, agenesis of a liver lobe, and absence of its segments are also other reported liver anomalies [5, 6]. The most common liver anomalies are irregularities in the shape of the liver and irregularities in the number of liver lobules. However, rarely, it may have accessory lobes; this anomaly has an estimated prevalence of less than 1%. An accessory lobe of the liver is congenital ectopic hepatic tissue mostly due to embryonic heteroplasia, although in rare instances it may occur after trauma or surgery [7]. One of the abnormalities reported several times is Riedel’s lobe [8], defined as a downward tongue-like projection of the anterior edge of the right liver lobe to the right of the gallbladder. The accessory lobes may be attached to the liver through a mesentery or a bridge of the hepatic tissue and are usually asymptomatic [1, 7, 9, 10].

The testes are a pair of oval-shaped male genital organs suspended in the scrotum by the corresponding spermatic cord. Undescended testis (cryptorchidism), discovered for the first time by Hunter in 1786, is a common anomaly encountered in pediatric urology [11].

The testes develop before birth in the dorsal abdominal wall. Testicular descent to the scrotum is not a simple process but appears to be multistaged, with various anatomical factors and hormonal influences [12–14]. Testes that are undescended at birth may descend spontaneously during early life, but seldom thereafter; by 12 months of age, approximately 1% of all boys have cryptorchidism [15, 16]. Testes may get arrested anywhere along the course to reach into the scrotum, such as in the abdomen, at the deep inguinal ring, in the inguinal canal, or between the superficial inguinal ring and the scrotum [1]. The incidence of undescended testis is around 0.8–2% in full-term newborns and around 18–30% in premature births [17]. The anomaly may be unilateral or bilateral; a unilateral undescended testis is present in 3% of the cases at birth and 1% at 3 months of age. On the other hand, the bilateral maldescent of testes was detected in approximately 1% of males at birth [18]. Anomalies of descent include cryptorchidism (anorchism, monorchism, and partially descended testis), ectopic testis, and persistence of processus vaginalis and encysted hydrocele of the spermatic cord [1]. The incidence of testicular cancer in patients with cryptorchidism is estimated to be 3 to 5 times higher than in the general population [10].

Detection of the syndromes associated with the anomalies and early intervention on the clinical cases allow prevention of adverse consequences like infertility and testicular carcinoma.

Overall, knowledge and awareness of the various anatomical variations have clinical significance to students, researchers, surgeons, and radiologists who interpret plain and computed imaging. Therefore, the main purpose of this case report is to demonstrate anatomical variation of anatomical structures such as additional liver lobe and testicular descent.

## Case presentation

During a gross anatomy dissection session for first-year medical students held at the Department of Human Anatomy, College of Medicine and Health Sciences, Bahir Dar University, two anatomical variations were noticed in an unclaimed male cadaver used for a routine teaching and learning purpose. The cadaver was obtained from the Amhara regional state in Ethiopia; the Amhara male cadaver was approximately 41-year-old and his clinical history, family history, and other details were unknown because it was an unclaimed cadaver. The hospital stated that the cause of death was not associated with the presented variations. Pictures of the cadaver were taken using digital cameras and are described in the next subsections.

### Dissection procedures

The dissection was performed, as usual, according to *Cunningham’s Manual of Practical Anatomy*, *Volume II* [19], for first-year preclinical medical students in the dissection room at the College of Medicine and Health Sciences of Bahir Dar University.

### Variation 1

**Fig. 1:**
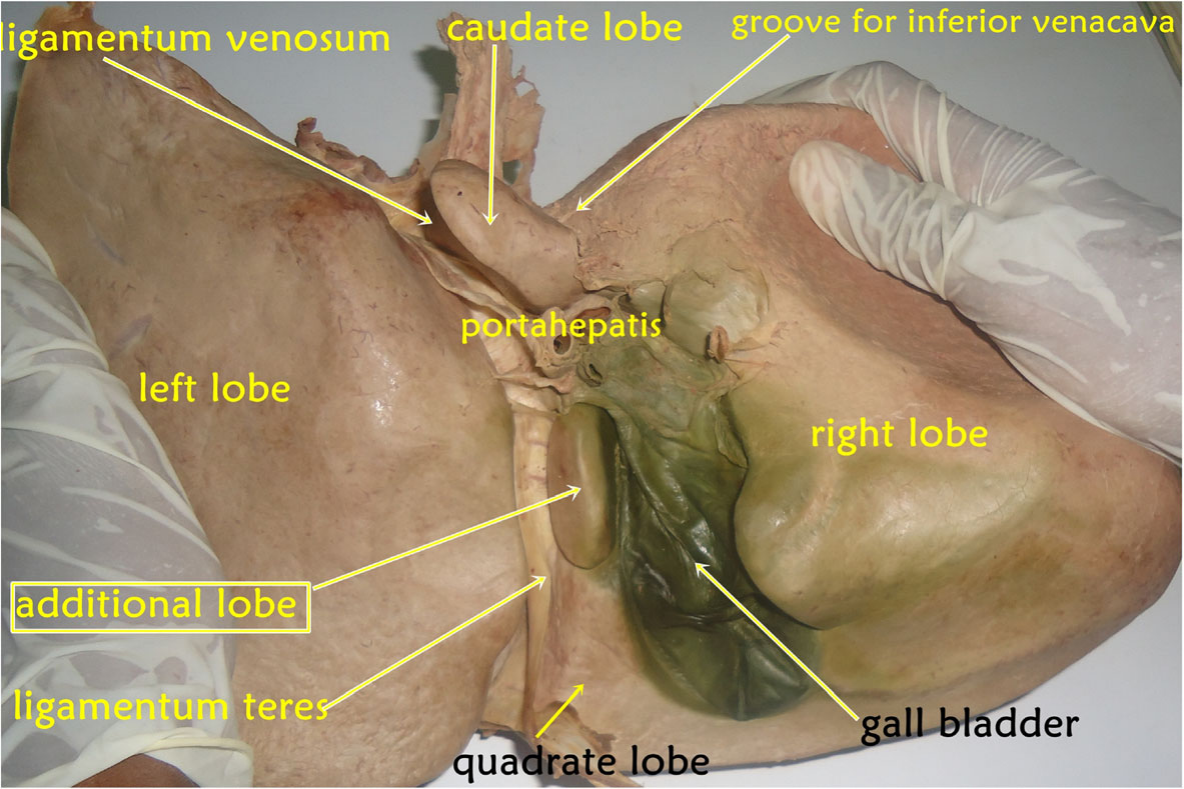
A photograph that was taken of the cadaver showing the liver as seen from its visceral surface, presenting its different impressions, structures and the additional lobe which is not usual in most liver specimens

To dissect the abdominal region, the cadaver was positioned in the supine position and the abdominal wall and cavity along with the peritoneum were dissected. After the reflection of the anterolateral abdominal wall and removal of the peritoneum, abdominal organs were observed for their anatomical locations in the abdominal cavity, and nothing new was observed on the diaphragmatic surface of the liver. On the other hand, during the detailed observation and study of each abdominal viscus by students and demonstration by the teachers, the liver was found to have anatomical variation; unusually the liver consisted of one additional (accessory) lobe situated on the visceral surface bounded by the following anatomical structures: to the right it was bounded by gall bladder; the ligamentum teres hepatis bound it on the left; and anteriorly it was demarcated by quadrate lobe of liver and posteriorly by porta hepatis of liver (Fig. 1).

### Variation 2

**Fig. 2:**
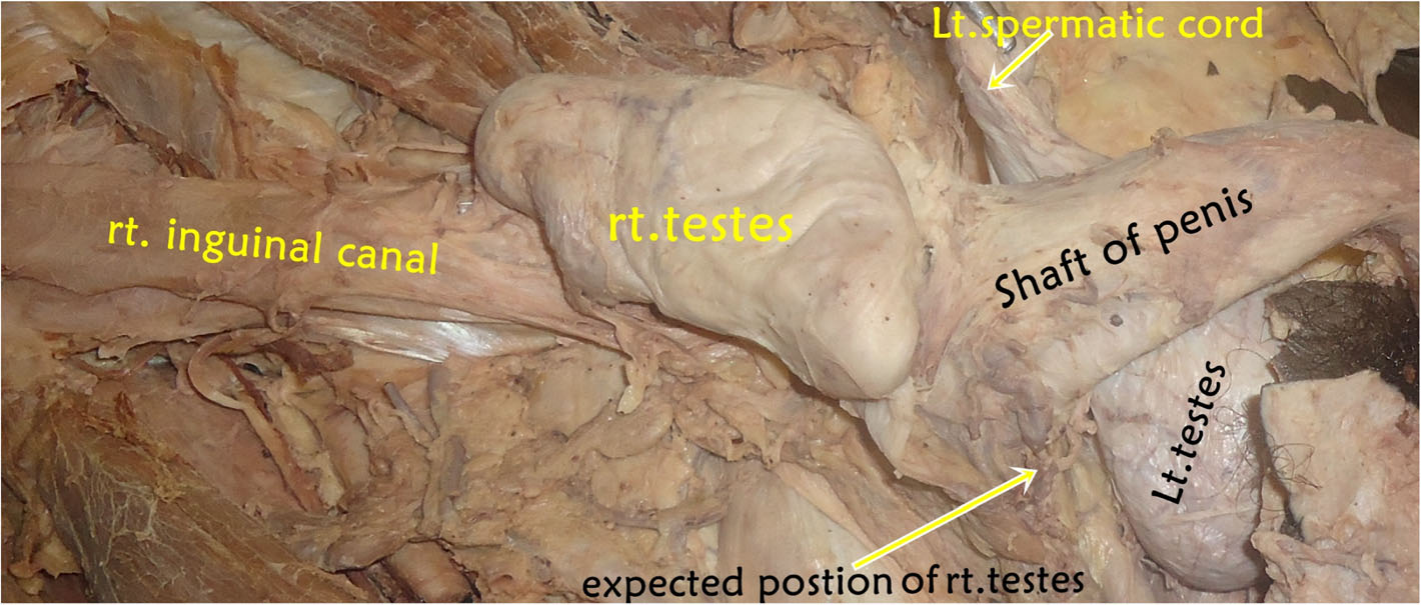
A photograph that was taken of the cadaver showing the ectopic location of the right testis out of the right scrotum. *rt*. right, *Lt*. left

The dissection lesson continued and the lower part of the abdominal wall (inguinal canal) was dissected and the spermatic cord was examined. The anatomy of the spermatic cord on the left side was as usual (normal). However, on the right side, the testis did not completely descend to the scrotum; instead, it was situated immediately distal to the opening of the superficial inguinal ring. As a result, the right spermatic cord became very short and was found empty up to the opening of the scrotum (Fig. 2).

## Discussion

Knowledge of the embryological development of organs is important to appropriately describe most of the congenital anomalies associated with the organs. As a type of congenital anatomical malformation, accessory lobes of the liver occur very rarely because the malformation is associated with an autosomal recessive gene with a very low frequency [20]. Classical textbooks of embryology describe liver development as follows: the liver develops from an endodermal hepatic bud that arises from the ventral aspect of the distal part of the foregut in the middle of the third week. The hepatic bud grows into the ventral mesogastrium and septum transversum. The bud soon divides into two parts: a large cranial part, pars hepatica, and a small caudal part, pars cystica. The pars hepatica forms the liver, while pars cystica forms the gallbladder and cystic duct. Initially, both lobes of the liver are of equal size. As the right and left portions of the pars hepatica enlarge, they extend into the septum transversum. The cells arising from these form interlacing hepatic cords or cords of hepatocytes. In this process, vitelline and umbilical veins present within the septum transversum get absorbed and broken to form the liver sinusoids. The cells of hepatic cords later become radially arranged in hepatic lobules. The bile canaliculi and ductules are formed in liver parenchyma and establish connections with extrahepatic bile ducts secondarily at a later stage [21–23]. Consequently, during this developmental process, the liver can present a number of congenital anomalies [1]. The commonest incidences are irregularities in the shape of the liver and irregularities in the number of lobules. However, an accessory liver lobe is a very rare occurrence and it is clinically important.

Netter and Colacino classified liver lobes into the following types: type 1 is very small left lobe and deep costal impressions, it occurred in 2% of specimens; type 2 is complete atrophy of left lobe, which occurred in 0% of specimens; type 3 is a transverse saddle-like liver and relatively large left lobe, which occurred in 10% of specimens; type 4 is a tongue-like process of the right lobe, which occurred in 2% of specimens; type 5 is a very deep renal impression and corset constriction in 2% of specimens; and type 6 is diaphragmatic grooves, which occurred in 2% of specimens [24].

Accordingly, in this cadaveric report, we observe that the liver contained one additional (accessory) lobe located between the quadrate and caudate lobes visible in its visceral surface; this observation does not match the above classifications. A study conducted in India reported a small accessory lobe of the liver in an adult male cadaver, which was situated in the posterior part of the fissure for ligamentum teres, close to the porta hepatis [1]. An adult cadaveric study on normal morphological variation of liver lobes using 50 specimens found accessory liver lobes in 8 cases (16%) [2]. Nayak [1] identified additional liver lobes in approximately 9% of cases among 55 livers of South Indian cadavers.

According to classical textbooks of embryology, testicular development and descent from the abdomen to the scrotum is a complex and multistage process that starts from 7th to 35th week of gestation. Normally, the testis follows the course of scrotal extension of gubernaculum, but, occasionally, it follows one of the other tails of the gubernaculum to an ectopic location in the perineum, suprapubic, femoral, or contralateral hemiscrotal areas [1, 21–23, 25]. Any disturbance in this process leads to a maldescent that could be in its normal pathway (true undescended testis) or an abnormal pathway (ectopic testis). Unilateral undescended testis is more likely to occur because androgens act independently on each side via the ipsilateral genitofemoral nerve [26].

In the present case (report), the right testis was found just distal to the superficial inguinal ring and the right compartment of the scrotum was totally empty (Fig. 2). Similarly, a recent study reported unilateral undescended (cryptorchidism) right and left testes situated at the superficial inguinal ring [1] and deep inguinal ring [12], respectively. Shankar and Kulkarni also declared the existence of unusual bilateral undescended testes engaged at superficial inguinal ring among the Indian population [16]. A rare encounter in pediatric surgical practice, unilateral (right testis) and bilateral perineal ectopic testis was experienced in Pakistan [25]. A prevalence study conducted at the University Teaching Hospital in Lusaka on 384 deceased adults showed that only one deceased adult presented with a right-sided impalpable testis [27]. In a study by Onkar *et al.* about undescended testes using high-frequency ultrasound which incorporated 41 boys, 30 patients had unilateral and 11 had bilateral undescended testes [28]. The prevalence was more on the right side (16 out of 30) and 26 (63%) were located in the inguinal canal [28].

In general, the occurrence of these two variations in a single cadaver has not been reported before, even though there have been reports of individual cases. There is also no developmental (embryological) background for the liver congenital anomaly to be associated with the undescended testis (cryptorchidism) and vice versa; therefore, this case is, by far, a coincidence.

## Conclusion

These two variations demonstrate that the classical textbook description of anatomical structures may not appropriately describe embryologic anomalies. This report adds substantial information to existing knowledge about the anatomical structures described in this case. Knowledge of the embryological development of structures is important to describe abnormalities. Therefore, students, researchers, embryologists, surgeons, and other concerned professionals should be aware of it.

## Data Availability

Not applicable.
